# The psychotherapist’s role in identifying and supporting mothers with postnatal depression: From detection to empowerment

**DOI:** 10.1192/j.eurpsy.2026.11391

**Published:** 2026-07-31

**Authors:** C. V. Valeriu

**Affiliations:** Department of Mental Health, Medical Psychology and Psychotherapy, Nicolae Testemițanu State University of Medicine and Pharmacy, Chisinau, Moldova, Republic of

## Abstract

**Introduction:**

Postnatal depression (PND) is often concealed by norms of joy and competence. About 10–20% of new mothers are affected, yet many are neither screened nor referred. Psychotherapists can detect shame- and guilt-laden distress and coordinate medical, emotional, and relational care.

**Objectives:**

To define the psychotherapist’s role in detecting PND and to outline a support model that fosters maternal empowerment—agency, meaning, and relational efficacy—beyond symptom relief.

**Methods:**

PRISMA 2020 systematic narrative review of MEDLINE, Embase, PsycINFO, Web of Science, Cochrane Library, and Scopus from the earliest records to 31 December 2024, no language limits. Eligible studies enrolled women ≤12 months postpartum, used validated tools (EPDS, PHQ-9, DSM/ICD), and reported therapist-led detection or psychotherapeutic support with outcomes on depression, bonding, alliance/engagement, or empowerment. Designs included RCTs, quasi-experimental, cohort, and mixed-methods. Risk of bias: RoB 2/ROBINS-I/CASP; certainty considered with GRADE. Semi-structured interviews with 30 perinatal psychotherapists underwent reflexive thematic analysis.

**Results:**

Embedding a validated screen (especially EPDS) at first psychotherapy contact increased case identification and referral completion versus symptom-led judgment. Early psychotherapy (≤6 months postpartum) produced small-to-moderate symptom reductions (typical SMD ≈0.3–0.6) and improved maternal–infant bonding and partner communication. Tele-enabled care matched in-person outcomes when integrated in structured screening-to-referral pathways. Clinicians described empowerment as the shift from global defectiveness to specific, repairable themes, supported by empathic validation, normalization of ambivalence, reflective dialogue, and gentle body-awareness. Barriers included stigma, time constraints, and fragmented referral loops.

**Conclusions:**

Psychotherapists are pivotal mediators from hidden suffering to recovery. Pairing validated early screening with attuned, attachment-informed psychotherapy improves detection, reduces symptoms, and nurtures empowerment. Services should normalize EPDS-based intake screening, formalize stepped referrals, and resource brief, group, and tele-enabled interventions for timely, equitable postpartum mental-health care.

**Disclosure of Interest:**

None Declared

